# Effects of gut microbiota in breast cancer

**DOI:** 10.3389/fonc.2025.1617410

**Published:** 2025-06-17

**Authors:** Leping Kang, Haiyang Chen, Chengjun He, Junchuan Li

**Affiliations:** ^1^ Department of Thyroid and Breast Surgery, Central Hospital of Ziyang, Sichuan, Ziyang, China; ^2^ Department of hepatobiliary and pancreatic Surgery, Central Hospital of Ziyang, Sichuan, Ziyang, China; ^3^ Department of General Surgery, Yanjiang District people’s Hospital of Ziyang, Sichuan, Ziyang, China

**Keywords:** breast cancer, gut microbiota, prebiotics, probiotic, dysbiosis

## Abstract

Breast cancer (BC) stands as the predominant form of cancer affecting women globally, with its etiology rooted in a complex interplay of factors. Emerging research underscores the significant impact of microbiota on the development of BC. Evidence points to a correlation between BC and microbial imbalance, not only within the intestinal milieu but also in breast tissue itself. Alterations in the diversity and functionality of bacterial populations in these areas are implicated in the disease’s pathogenesis.The intestinal microbiota exerts a pivotal influence on the metabolism of steroid hormones, including estrogens, which are recognized as critical elements in the risk profile for BC, particularly among women who have reached menopause. These hormones can drive the onset and advancement of BC via multiple mechanisms. Concurrently, a body of research highlights the contributory roles of immune system elements, inflammatory processes, dietary patterns, and the use of probiotics in the context of BC. Understanding this intricate interplay holds promise for developing innovative therapeutic approaches

## Introduce

Breast cancer (BC), representing 32% of newly diagnosed cancer cases, stands as one of the most prevalent malignancies among women globally. Despite substantial advancements in diagnostic and therapeutic approaches, the five-year survival rate for BC patients averages 89.2%, with tumor stage being a critical determinant of disease progression (1). Specifically, the survival rate exceeds 98% for stage I tumors but drops significantly to 24% for stage III tumors. While factors such as diet, alcohol consumption, and radiation exposure have been linked to an elevated risk of BC, emerging research highlights the potential role of the human microbiome in influencing this disease (2, 3). Both the gut microbiota and the local microbiota within the mammary gland have been proposed to impact BC progression. This review aims to elucidate the mechanisms through which the gut microbiota may contribute to cancer development, with a particular focus on BC, and to summarize clinical trials investigating alterations or modifications in the microbiota of women diagnosed with BC (4–6).

## General situation

For a long time, the mammary gland was assumed to be a sterile environment. However, current research has revealed the presence of a distinct microbiota within it (7). Structurally, the breast mainly consists of adipose tissue, complemented by an extensive network of blood vessels and lymphatic drainage. This unique physiological makeup creates a favorable ecological niche for the growth of bacteria, particularly Proteobacteria and Firmicutes (8). Through culture - based experiments, the existence of viable bacteria in the mammary tissue has been conclusively demonstrated. Among the identified species are Bacillus sp., Enterobacteriaceae sp., and Staphylococcus sp (7). In a study involving Asian BC patients, researchers discovered an enrichment of Propionicimonas, Micrococcaceae, Caulobacteraceae, Rhodobacteraceae, Nocardioidaceae, and Methylobacteriaceae within tumor tissues (9). In general, BC patients often exhibit a reduction in microbial diversity, accompanied by significant changes in the composition of their microbiota (10). In a separate clinical trial, Luu et al. observed substantial differences in the absolute counts of total bacteria, Firmicutes, Faecalibacterium prausnitzii, and Blautia in fecal samples. Intriguingly, these findings were closely correlated with the body mass index (BMI) of women with early - stage BC. Specifically, overweight and obese patients were found to have a lower bacterial count (11).Consistent with these findings, other research groups have reported that BC patients typically have a lower relative abundance of Firmicutes and Bacteroidetes in their feces, while simultaneously showing an elevated relative abundance of Verrucomicrobia and Proteobacteria (12). Frugé et al., in their investigation of early - stage BC patients, found that body composition was inversely associated with the abundance of Akkermansia muciniphila. Conversely, it was positively correlated with interleukin - 6 levels. Additionally, they reported that the relative abundance of Akkermansia muciniphila was linked to key health - related outcome parameters and was associated with beneficial dietary modifications (13).In a comparative study of breast tissue samples from BC patients and healthy controls, Propionibacterium and Staphylococcus were found to be depleted in tumor tissues. This depletion was negatively associated with oncogenic immune features. On the other hand, Streptococcus and Propionibacterium were positively correlated with genes related to T - cell activation (14). Costantini’s research identified Proteobacteria, Firmicutes, Actinobacteria, and Bacteroidetes as being associated with breast tumors, with Ralstonia being the most dominant genus among them (15). Meng, using a Chinese patient cohort, observed an increased representation of the genus Propionicimonas, as well as the families Micrococcaceae, Caulobacteraceae, Rhodobacteraceae, Nocardioidaceae, and Methylobacteriaceae in malignant breast tumor tissues. Nevertheless, it is essential to acknowledge that these results may be influenced by the ethnic - specific characteristics inherent to the studied population (16). Differences in the urine microbiota of BC patients have also been identified. These differences are characterized by an increased presence of gram - positive organisms, including Corynebacterium, Staphylococcus, Actinomyces, and Propionibacteriaceae, and a concurrent decrease in the abundance of Lactobacillus. Nejman et al. further demonstrated that breast tumor samples had a higher bacterial load and species richness compared to breast samples obtained from healthy subjects within the studied population (17). Notably, in women with the HER2+ subtype of BC, which is known for its aggressive growth and rapid spread, there was a decrease in fecal α - diversity and Firmicutes abundance, while the abundance of Bacteroidetes was found to be elevated (18).

## Estrogen and microbiota

Exposure to hormones ranks as one of the most significant factors linked to the development of BC, especially among postmenopausal women. In this context, the concept of the estrobolome is of particular significance. The estrobolome encompasses a set of microbial genes whose products are actively involved in the complex process of estrogen metabolism (19). Numerous studies have convincingly demonstrated that a specific subset of microorganisms residing within the gastrointestinal tract exerts a notable influence on estrogen metabolism. This influence extends to regulating the delicate balance between circulating estrogen levels in the bloodstream and those excreted from the body (20). More specifically, free estrogens are predominantly generated through a deconjugation process that occurs within the gut environment. This process is facilitated by the enzyme bacterial β - glucuronidase, which is particularly active within microbial communities such as those belonging to the Clostridia and Ruminococcaceae families, or the Escherichia/Shigella genus (21, 22). The journey of estrogen metabolism commences in the liver. Here, estrogens undergo conjugation reactions, after which they are excreted into the gastrointestinal lumen as part of the bile. Once in the gut, the conjugated estrogens encounter bacterial β - glucuronidase, which breaks them down through de - conjugation. The resulting free estrogens are then re - absorbed into the bloodstream through the enterohepatic circulation. This process allows the free estrogens to reach various organs throughout the body, with the breast being one of the key destinations (23–25). In addition to its role in estrogen metabolism, β - glucuronidase may also play a major part in the deconjugation of endocrine - disrupting chemicals. These chemicals can disrupt the normal composition and function of the gut microbiota. Moreover, they can also alter the metabolites produced by these microorganisms, potentially leading to an increase in inflammation within the body (26). Therefore, any perturbations or imbalances in the microbiota - estrobolome axis can lead to an elevation in the levels of circulating estrogens and their metabolites. This increase in estrogenic activity is a well - recognized risk factor for the development of BC, as it can potentially stimulate the growth and proliferation of BC cells.

Available literature indicates that postmenopausal women with BC exhibit increased levels of *Clostridiaceae* in their fecal microbiota (27). However, a study by Angioletta reported no significant disparities in microbial profiles between patients experiencing disease relapse during aromatase inhibitor therapy and relapse-free control subjects. Instead, this investigation highlighted that the *Clostridia* class and *Clostridiales* order represented the most abundant taxonomic groups within the case cohort. Notably, the *Veillonella* family, which was enriched in BC cases, possesses the capacity to synthesize β-glucuronidase—an enzyme implicated in augmenting free estrogen levels through deconjugation reactions (28).

A diverse array of bacterial taxa are capable of synthesizing β-glucuronidase, including genera such as *Alistipes*, *Bacteroides*, *Bifidobacterium*, *Collinsella*, *Edwardsiella*, and *Faecalibacterium*, alongside specific species within the *Lactobacillus* and *Roseburia* clades (29, 30). Given the pivotal role of the gut microbiota in modulating intratumoral inflammatory and immune microenvironments, variations in microbial composition—including shifts between eubiosis and dysbiosis—are hypothesized to influence the clinical response to hormone therapy (31). Additionally, although direct evidence remains lacking, a mechanistic link is proposed whereby microbiota-mediated elevations in estrogen bioavailability or enzymatic degradation of orally administered hormonal agents may alter drug pharmacokinetics, thereby impacting therapeutic efficacy.Accumulating evidence indicates that certain gut-resident bacterial species encode enzymes capable of modulating the enterohepatic circulation of estrogen metabolites, facilitating conversions of estrogens to androgens or biosynthesis of estrogen-mimetic molecules (32). Preclinical studies in murine models have shown that prolonged estrogen supplementation disrupts gut microbiota composition—specifically leading to a decrease in *Akkermansia muciniphila* abundance—and alters β-glucuronidase activity, collectively indicating a bidirectional interaction where estrogen homeostasis influences microbiome dynamics (33).

## Direct action

Live bacteria have been identified within tumor cells and tumor-associated immune cells (4, 17), suggesting that cancerous and host cells may serve as carriers to facilitate the spread of microorganisms to tumors or adjacent normal mammary tissues (34). In the context of BC, Parida et al. demonstrated that gut colonization with enterotoxigenic *Bacteroides fragilis* (ETBF), which secretes *B. fragilis* toxin (BFT), induces epithelial hyperplasia in the mammary gland. Furthermore, *in vitro* treatment of BC MCF7 cells with BFT prior to injection in mice significantly accelerated tumor growth and metastasis through the β-catenin and Notch1 signaling pathways (19). Gut-resident bacteria may translocate to the mammary gland via breaches in the intestinal epithelium, a common occurrence during dysbiosis, and travel through the bloodstream or lymphatic system. Alternatively, intestinal dendritic cells, which can penetrate tight junctions between epithelial cells to uptake bacteria, may transport these microorganisms to distant sites, including mammary tissue, via the vascular system (35). It has been hypothesized that alterations in bacterial abundance or composition within tumors may result from the disease itself, as the leaky vasculature of the tumor microenvironment could facilitate bacterial recruitment (36). Urbaniak et al. investigated the ability of bacterial isolates from adjacent normal tissue of BC patients to induce DNA double-stranded breaks (37). Additionally, nisin, a bacteriocin produced by the Gram-positive *Lactococcus lactis*, has demonstrated potent cytotoxic effects on breast tumor cells by disrupting calcium ion influx and inducing cell cycle arrest (38). Another example is *Bacteroides fragilis*, a gut bacterium also found in the mammary gland, whose toxin can promote epithelial hyperplasia, tumor growth, and metastasis via the β-catenin–Notch1 axis (19). Furthermore, *Fusobacterium nucleatum*, previously linked to colorectal cancer, has been implicated in BC progression. This bacterium activates the TLR4/NF-κB pathway in cancer cells (39–42) and, through its Fap2 lectin protein, binds to Gal-GalNAc, a sugar highly expressed on breast tumor cells. This interaction accelerates tumor growth and metastasis (43, 44).

## Immune system

Initially, BC was thought to be a non-immunogenic tumor. However, recent studies have revealed that the expression of immune-related genes and the presence of immune infiltrates in primary tumors are associated with improved clinical outcomes (45). The gut microbiota influences the immune system of BC patients by regulating the proliferation and differentiation of regulatory T cells, inducing the expression of secretory immunoglobulin A (sIgA), and modulating neutrophil production. Reduced microbiome diversity has been linked to poorer survival rates in BC patients, suggesting a critical role for microbial balance in disease progression. Researchers have proposed that gut microbiota dysbiosis can disrupt the host immune system, with IgA protein serving as a potential link between BC-related inflammation and gut microbiota (46). A 2018 case-control study found that IgA+ patients exhibited significantly lower microbiota abundance and α-diversity compared to IgA- patients, indicating that gut microbiota may influence BC development by altering immune pathways (47). These immune alterations are often accompanied by changes in immune cell populations, including decreased lymphocyte levels and increased neutrophil counts (48). Neutrophils, in particular, have been shown to be influenced by gut microbiota in the context of BC. For example, in the C3-1-TAg mammary cancer mouse model, infection with *Helicobacter hepaticus* (a gut-resident bacterium) promoted BC progression, correlating with increased neutrophil recruitment and infiltration at the tumor site (49). Additionally, M2-like macrophages, the predominant immune subset in the breast tumor microenvironment, are associated with reduced survival in hormone receptor-positive (HR+) BC. These macrophages infiltrate both breast tumors and adjacent normal mammary tissues during early and advanced stages of tumor progression (50, 51).

Furthermore, specific gut bacteria have been shown to modulate the immune response in a way that either promotes or suppresses tumor development, largely through the regulation of stimulator of interferon genes (STING) agonists. For instance, the presence of *Akkermansia muciniphila*, a gut bacterium that produces cyclic di-AMP (cdAMP), has been observed to activate the STING pathway, leading to the induction of the type I interferon (IFN-I) pathway. This IFN-I production reprograms macrophages into an anti-tumor phenotype and enhances the crosstalk between natural killer (NK) cells and dendritic cells (DCs), thereby fostering a robust anti-tumor immune response (52). Zubaida et al. explored the anti-cancer potential of heat-killed cells (HKC) and cytoplasmic fractions (CF) of *Enterococcus faecalis* and *Staphylococcus hominis* in the MCF-7 BC cell line. Both bacterial forms significantly reduced MCF-7 cell proliferation, induced apoptosis, and caused cell cycle arrest in a concentration- and time-dependent manner (53). The presence of these bacteria was also positively correlated with the production of trimethylamine N-oxide (TMAO), a metabolite known to activate CD8+ T cell-mediated anti-tumor immunity and promote M1 macrophage polarization. This further supports the concept of a metabolically active, tissue-resident microbiota playing a role in cancer immunity (54). In a recent study involving triple-negative BC patients, a high abundance of *Clostridiales* in tumor tissue was associated with an activated immune microenvironment (55). Additionally, *Sphingomonas*, a bacterium detected in healthy mammary tissue, has been shown to activate invariant natural killer T (iNKT) cells (56), which are critical mediators of cancer immunosurveillance and the control of BC metastases (54). Notably, higher levels of *Sphingomonas* in healthy mammary tissue compared to tumor tissue have been linked to increased expression of Toll-like receptors (TLR2, TLR5, and TLR9) and antimicrobial effectors such as IL-12A, bactericidal/permeability-increasing protein (BPI), and myeloperoxidase (MPO). This suggests a potential protective role of *Sphingomonas* in cancer by enhancing immunosurveillance (57).

Emerging evidence suggests that shifts in the abundance of specific gut microbiota can modulate immune responses by increasing the production of regulatory T cells (Tregs) or reducing the differentiation of pathogenic T cells, potentially mitigating inflammatory diseases (58). Collectively, these findings highlight the role of microbial DNA and bacterial metabolites in shaping the local immune microenvironment within the breast. This implies that commensal bacteria may directly influence tumor progression through their metabolic activity, which can alter immune cell behavior and regulate inflammatory processes.

## Inflammation and microbiota

Inflammation is a hallmark of carcinogenesis, irrespective of the underlying cause, and is considered a primary oncogenic mechanism driven by the microbiota (59). Microbial virulence factors can trigger chronic inflammation in host tissues, promoting excessive cell proliferation. When this proliferation becomes dysregulated and is coupled with impaired apoptosis, it can initiate the carcinogenic process (60, 61).

Certain harmful microbes in the human gut, like Escherichia coli and Serrella, are able to heighten chronic inflammation and autoimmune reactions. They do this either by generating endotoxins or by stimulating the differentiation and function of Th17 cells (62). Gut bacteria can contribute to BC development through chronic inflammation, which is closely tied to tumorigenesis. These bacteria can cause an upregulation of Toll - like receptors (TLR) and activate NF - κB. NF - κB is vital for inflammation regulation and has a connection with cancer. Once activated, it leads to the release of cytokines such as IL - 6, IL - 12, IL - 17 and IL - 18. This, in turn, triggers ongoing inflammation within the tumor microenvironment (63–65). Inflammation triggered by pathogens isn’t restricted to the infection site. For instance, C57BL/6 ApcMin/+ mice, which have a genetic tendency to develop breast carcinomas, won’t develop breast tumors if they’re raised in a specific pathogen - free environment (66). But when Helicobacter hepaticus is administered to them via the stomach, they develop mammary carcinomas because of the activation of the innate immune response through inflammation (67, 68). Conversely, beneficial gut microbes such as bifidobacterium and lactic acid bacteria can boost the production of short - chain fatty acids (SCFAs). The increased SCFAs can promote the differentiation and function of Tregs. As a result, chronic inflammation and autoimmune responses are inhibited (69).

As highlighted by Angioletta, estrogens exert anti-inflammatory effects by directly modulating CD16 expression, thereby reducing pro-inflammatory IL-6 (70). Concurrently, these sex hormones are shown to influence the homeostasis of NK cell populations, underscoring their dual role in immune regulation and inflammation control.Available evidence from these investigations indicates that specific antimicrobial agents may diminish inflammatory mediators—at least in the short term—by enhancing the excretion of conjugated estrogens, potentially lowering the risk of BC through this mechanistic pathway (21). However, the long-term effects of antibiotic use on estrogen elimination dynamics and associated cancer risk remain unexplored, representing a critical knowledge gap in this field.

Thus, it can be concluded that chronic inflammation plays a critical role in both the initiation and progression of BC. The persistent presence of inflammatory cytokines and the recruitment of immune cells, such as Tregs, contribute to a suppressed immune response, facilitating tumor immune escape and promoting cancer progression.

## Diet and microbiota

Dietary changes are well-documented to influence the composition and function of the gut microbiome (62). Approximately 35% of all cancers, including 50% of breast carcinomas, are linked to dietary factors (63). In diet-associated BC, microbial-mediated mechanisms are thought to play a role in modulating carcinogenesis and tumor aggressiveness (64). Diets rich in mono- and polyunsaturated fatty acids, gut microbiota-accessible carbohydrates, fruits, vegetables, and legumes are associated with improved overall health and may offer protective effects against cancer risk and mortality, including BC (65, 66). Hypercholesterolemia has been identified as a risk factor for estrogen receptor (ER)-positive BC (67). The cholesterol metabolite 27-hydroxycholesterol (27HC) exhibits estrogenic activity and has been shown to promote breast tumor growth in xenograft mouse models by binding to ERs on mammary gland epithelial cells and stimulating proliferation (68). However, its role in humans remains to be fully elucidated (69).The mechanistic link between alcohol consumption and BC may involve several pathways: 1) alcohol may enhance ER signaling in breast tumors or elevate endogenous steroid hormone levels (71); 2) ethanol can stimulate the transcriptional activity of ER-α in human BC cell lines in a dose-dependent manner while downregulating *BRCA1*, a gene that inhibits ER-α transcriptional activity (72); and 3) in healthy postmenopausal women not on hormone replacement therapy (HRT), daily alcohol consumption of 15–30 g was associated with a 7.5% and 10.7% increase in serum estrone sulfate levels, respectively, compared to non-drinkers (71).

## Probiotics and prebiotics on microbiota

Probiotics are live microorganisms that help maintain a healthy gut microbiota and restore beneficial microbial balance (73). Research by Imani Fooladi demonstrated that treatment with *Lactobacillus acidophilus* significantly extended the survival time of BC mice compared to a control group (74). Members of the *Lactobacillus* and *Bifidobacterium* genera exhibit therapeutic potential in shaping the tumor microenvironment through dual mechanisms: regulating cytokine profiles and augmenting the activity of NK cells alongside cytotoxic T lymphocytes (CTLs) (75). One of the key features of probiotics is their ability to produce beneficial compounds, such as antibiotics, anticarcinogens, and other substances with positive effects on overall health and pharmaceutical properties (76). Epidemiological studies have linked the consumption of fermented dairy products to a reduced risk of BC, potentially due to changes in the gut microbiota that alter the enterohepatic cycling of estrogenic compounds. Marschalek et al. found that oral probiotic formulations could improve the vaginal microbiota in postmenopausal BC patients undergoing chemotherapy, highlighting a potential therapeutic application of probiotics in BC management (77). Further supporting this, Imani Fooladi et al. showed that daily oral administration of *L. acidophilus* two weeks before BC tumor transplantation and continued for 30 days significantly increased overall survival, suggesting that *L. acidophilus* may enhance immune responses and boost anti-tumor activity (74). Clinical trials and retrospective analysis have also demonstrated that probiotics can improve quality of life, reduce therapy-related toxicities, and mitigate complications in cancer patients (75). For example, in Japanese women, regular consumption of *Lactobacillus casei* Shirota and soy isoflavones from adolescence was associated with a reduced risk of BC, indicating potential chemopreventive effects (78). Chung introduced a innovative strategy for bacterial-mediated delivery of therapeutic proteins to colorectal cancer lesions, leveraging metabolic pathway rewiring to overcome promoter-associated limitations and enhance treatment precision and efficacy (79), but the translational potential of this approach to BC remains unestablished and warrants further preclinical validation.Concurrently, Prasoon highlighted the emerging role of artificial intelligence (AI)-driven synthetic biology methodologies in optimizing the therapeutic and nutritional attributes of probiotics (80). This technological paradigm shift is poised to revolutionize microbial engineering by enabling precise modulation of probiotic functions, which may have far-reaching implications for cancer therapeutics and host-microbiota interactions.

Prebiotics are non-digestible dietary compounds that promote the growth or activity of beneficial gut microorganisms (81)[. Dietary fiber, a common prebiotic, can modulate the gut microbiota and influence estradiol metabolism by affecting specific enzyme activities, such as β-glucuronidase, particularly in postmenopausal BC patients (82). Zengul et al. investigated the interplay between dietary fiber and gut microbiota, focusing on their role in enhancing β-glucuronidase activity and increasing circulating estrogen levels in postmenopausal BC patients (83).

When the gut microbiota is in a state of equilibrium, known as eubiosis, it can serve as a protective factor against cancer. This is achieved through the fermentation of dietary fiber, which helps maintain the integrity of the intestinal mucosa and supports the immune system, potentially triggering anti-tumor immune responses. Preclinical studies have suggested that probiotics may act as moderators to prevent or control BC progression by enhancing the host’s immune system. However, further research through clinical trials and prospective studies is necessary to confirm the efficacy of probiotics in the clinical management of BC.

## Antibiotics and microbiota

The use of antibiotics in cancer patients is a highly debated topic. Antibiotics are often prescribed in conjunction with chemotherapy and cancer surgeries. In certain types of cancer, like BC and melanoma, it has been noted that antibiotics can speed up disease progression (84). A case - control study involving 2266 North American women with BC and 7953 healthy controls revealed that women with a history of long - term antibiotic treatment had an increased risk of developing BC (85). A recent meta - analysis indicated that the type of antibiotic might be linked to BC risk. Specifically, the risk was slightly higher when patients were treated with penicillin, tetracycline, and nitrofuran, and there was a marginal increase with the use of nitroimidazole and metronidazole (86). In a study using BC mouse models, the administration of antibiotics led to a decrease in fecal butyrate levels and an increase in tumor growth (87). However, more large - scale prospective studies are needed to better clarify the role of antibiotics as a biomarker and in cancer treatment (88).

## Fecal microbiota transplantation and AI

In an investigation by Xu, ovarian cancer cell xenografts in mice with intestinal microbiota dysbiosis exhibited enhanced tumor growth compared to control groups (89). This dysbiotic state activated macrophages, leading to elevated circulating levels of interleukin IL-6 and TNF-α, which in turn promoted epithelial-mesenchymal transition (EMT) in ovarian cancer cells.A separate study utilizing antibiotic-induced microbiota-depleted mice to model endometriosis progression revealed that microbial depletion attenuated endometriotic lesion growth (90). Reciprocally, oral gavage of fecal microbiota from endometriosis-bearing mice restored lesion expansion in MD recipients, indicating a causal role for gut microbial composition in disease progression. While FMT has emerged as a promising approach for treating various cancers—including colorectal, hepatic, and pancreatic malignancies, melanoma, Clostridioides difficile infection, and radiation enteritis—its application in BC remains underinvestigated (91–93). Although the precise molecular and cellular mechanisms underpinning FMT’s effects remain unclear, proposed pathways involve direct interactions between donor microbiota, host intestinal epithelium, and immune system (94). These interactions are hypothesized to modulate intestinal mucosal barrier integrity, inflammatory signaling, and anti-tumor immune responses, highlighting the need for targeted studies to elucidate FMT’s role in BC management.

Historically, investigations into the gut microbiome have predominantly relied on traditional techniques such as 16S rRNA sequencing. However, this approach is constrained by its limited taxonomic resolution and inability to characterize the functional activities of microbial communities, thereby restricting mechanistic insights into host-microbiota interactions. AI has increasingly been integrated into medical research and applications. In the context of gut microbiota studies, a key challenge in leveraging AI lies in integrating multi-omics datasets to characterize the intricate interactions between the microbiome and host systems—encompassing metabolic, immune, and neural pathways. To tackle this complexity, researchers are progressively adopting integrated multi-omics frameworks that combine genomic, transcriptomic, proteomic, and metabolomic data, enabling comprehensive modeling of host-microbiota interactions at multiple biological scales (95). AI-driven models have demonstrated utility in discriminating between healthy and dysbiotic microbiome profiles, enabling the identification of potential biomarkers for diseases such as inflammatory bowel disease, obesity, and colorectal cancer (96). Despite these advancements, Critical challenges include the need for large, rigorously annotated training datasets and the risk of model overfitting to cohort-specific biases, which compromise the generalizability of such approaches for common tumors—including BC (97).

## Conclusions

Research into the possible interactions between the gut microbiome and BC encompasses the entire translational research spectrum. This type of study calls for cooperation among various professionals. These include basic scientists like immunologists, who study the immune system; cell biologists, who focus on the structure and function of cells; and microbiologists, who deal with microorganisms. Clinicians such as oncologists, who treat cancer patients, and endocrinologists, who specialize in hormone - related disorders, are also crucial. Additionally, animal researchers, who conduct experiments on animals to understand biological processes, epidemiologists, who study the patterns and causes of diseases in populations, biostatisticians, who analyze biological data using statistical methods, and bioinformaticians, who manage and analyze biological data using computational tools, all need to work together.
